# Understanding the Episodic Memory and Executive Functioning Axis Impairment in MCI Patients: A Multicenter Study in Comparison with CSF Biomarkers

**DOI:** 10.3390/biomedicines11123147

**Published:** 2023-11-26

**Authors:** Brenda Chino, Lucía Torres-Simón, Agnieszka Żelwetro, Inmaculada Concepción Rodríguez-Rojo, Anna Carnes-Vendrell, Gerard Piñol-Ripoll, Raquel Yubero, Nuria Paúl, Fernando Maestú

**Affiliations:** 1 Institute of Neuroscience, Autonomous University of Barcelona (UAB), 08193 Barcelona, Spain; brendachvilca@gmail.com; 2 Center for Cognitive and Computational Neuroscience, Universidad Complutense de Madrid, 28040 Madrid, Spain; lucia.torres@ucm.es (L.T.-S.); fmaestuu@ucm.es (F.M.); 3 Department of Experimental Psychology, Cognitive Processes and Speech Therapy, Universidad Complutense de Madrid, 28040 Madrid, Spain; napaul@ucm.es; 4 Interdisciplinary Doctoral School, SWPS University of Social Sciences and Humanities, 53-238 Wrocław, Poland; azelwetro@gmail.com; 5 Alzheimer’s Disease Research, Center in Ścinawa, 59-330 Ścinawa, Poland; 6 Department of Nursing and Physiotherapy, Faculty of Medicine and Health Sciences, Universidad de Alcalá, 28801 Madrid, Spain; 7 Unitat de Trastorns Cognitius, Cognition and Behavior Study Group, Universitat de Lleida, IRBLleida, 25198 Lleida, Spain; acarnes@gss.cat (A.C.-V.); gerard_437302@hotmail.com (G.P.-R.); 8 Neurology Department, Hospital Quirónsalud Madrid, 28223 Madrid, Spain; ryubero@quironsalud.es; 9 Instituto de Investigación del Hospital Clínico San Carlos, 28040 Madrid, Spain

**Keywords:** CSF biomarkers, neuropsychology, test of memory strategies, executive functions, episodic memory, mild cognitive impairment

## Abstract

Background: This study aimed to explore the association between a verbal learning task that evaluates the potential mutual dependency between memory and executive functions (i.e., the Test of Memory Strategies, TMS) and cerebrospinal fluid (CSF) Alzheimer’s Disease (AD) biomarkers. Methods: A sample of 47 mild cognitive impairment (MCI) participants from Poland and Spain were classified according to the Erlangen Score Diagnostic Algorithm (ESA) into CSF- (*n* = 16) and CSF+ (*n* = 31) groups. Correlation analyses between TMS word-list conditions and CSF biomarkers were conducted. Additionally, an analysis of covariance was performed to define the effect on ESA classification in the sample, using as a covariable the country of origin of the participants. Results: Significant associations between the TMS-3 condition and Aβ42, t-tau, and p-tau were observed for the whole sample. In addition, the CSF- participants obtained higher cognitive performance in TMS-3 compared to the CSF+ group. This outcome persisted if the groups were based on Aβ42 scores, but not t-tau or p-tau values. Conclusions: These findings could indicate that poor performance on verbal learning tests may be affected by executive dysfunctions. Therefore, future intervention plans focused on training executive functions would be of interest to improve the ability of MCI patients to encode and organize information.

## 1. Introduction

In recent decades, the abrupt increment in life expectancy has led to an aging population. Nowadays, 9% of the worldwide population is over 65 years old, and the number of people aged 80 years or over is projected to triple from 143 million in 2019 to 426 million in 2050 [1]. This dramatic change in population structure raises the risk of age-related pathologies like dementia.

Alzheimer’s Disease (AD) is the major cause of clinical dementia in the elderly [2]. From a neuropathological perspective, it is well characterized by the accumulation of amyloid-β (Aβ) neuritic plaques, neurofibrillary tangles formed by hyperphosphorylated tau protein, and the loss of synapses [3,4]. Note that cerebrospinal fluid (CSF) has been stated as a sensible and validated biomarker for AD diagnosis [5,6,7]. For example, it is well reported and extensively replicated that lower CSF Aβ-42 concentrations and higher CSF levels of total and phosphorylated tau (t-tau and p-tau, respectively) are typical findings among AD and mild cognitive impairment (MCI) patients [5,8]. Additionally, the relationship between CSF values and the accumulation of amyloid and tau proteins in certain brain regions reported in amyloid Positron Emission Tomography (PET) has been already established [9]. Amyloid deposits are typically found in certain nodes of the default mode network (prefrontal and parietal regions), while tau is associated with medial and neocortical temporal regions in MCI patients [10]. Therefore, these two types of deposits affect different functional networks involving both memory and executive functions.

From a neuropsychological standpoint, there is typically an important source of confusion regarding the subjective and objective cognitive symptoms in the early stages of AD. Whether they are of executive function or episodic memory nature is still a matter of debate. The performance of classical episodic memory tests involves both cognitive functions, making it difficult to discriminate the origin of the cognitive decline. This is an important issue, as it has been established that episodic memory failure is a proxy for AD, while executive function impairment could serve as a predictor of other types of dementia [11,12]. In this sense, Yubero et al. [13] developed the Test of Memory Strategies (TMS) specifically for differentiating these two cognitive symptoms with a word-learning paradigm [13]. 

The TMS is formed by five word-list conditions, which allows clinicians to easily evaluate two cognitive constructs simultaneously: memory and executive functions. In this sense, conditions 2 to 5 (i.e., from low to high material organization strategies) progressively reduce the need to use executive functions towards evaluating the primary capacity for episodic memory (see Section 2.3. for a more detailed explanation of the test). Based on the TMS conditions, discriminant analysis revealed 90% sensitivity and specificity to discriminate between different neurocognitive disorders such as amnestic or multidomain MCI or vascular cognitive impairment patients [13]. These results indicated how executive functions influence performance on memory tasks in elderly subjects with different neuropsychological profiles. Furthermore, executive and memory functions have been established as independent factors through TMS performance in a sample of Portuguese elderly subjects [14] and middle-aged Italian healthy adults [15]. 

Numerous studies have invested considerable resources into investigating the relationship between CSF biomarkers and cognitive performance in order to improve the early discrimination of AD and MCI patients. For example, it has been observed that tau load is more directly correlated with cognitive decline than total amyloid plaque aggregates [16,17]. Additionally, CSF tau values seem to be more associated with memory performance, while lower Aβ-42 concentrations predict a faster conversion to dementia [18]. Nevertheless, when combining CSF biomarkers with neuropsychological performance, the findings have demonstrated a correlation between both biomarkers (i.e., higher CSF tau and lower Aβ-42 values) and a decline in global cognition and episodic memory [19,20]. 

In this vein, episodic memory deterioration has been closely related to an increased likelihood of developing AD in MCI patients [21,22]. However, failures in other cognitive processes, such as verbal fluency or executive functions, have also been associated with lower levels of Aβ-42 and very high values of tau and p-tau [23,24]. Given these CSF biomarker associations with different cognitive domains in classical neuropsychological tests, discriminative tasks such as TMS could help in the early differentiation of the underlying cause of cognitive impairment. However, up to this point, there is no research in the literature studying the possible relationship between TMS scores and one of the most clinically established biomarkers in AD (i.e., CSF tau and Aβ42 values). 

In order to understand the episodic memory and executive functioning impairment in MCI patients, we used the TMS. To comprehend its linkage to current biomarkers of AD, we computed a series of Spearman correlations between CSF values and TMS scores. Our hypothesis is that executive functioning impairment plays a pivotal role in the performance of verbal learning tasks, surpassing the previously reported significance for patients with MCI. Furthermore, we expect an improvement in the performance of verbal learning tasks when the use of executive functions is diminished. These phenomena would be closely related to the levels of proteins found in CSF. 

## 2. Materials and Methods

### 2.1. Participants

The sample comprised 135 Caucasian participants, aged between 61 and 85 years, who were both consecutively and prospectively recruited from a multicenter international study conducted in two health/research centers located in Spain (with 48 participants) and Poland (with 87 participants). The first one was the Cognitive Disorders Unit of the Hospital Universitario Santa Maria in Lleida, Spain, and the second was the Research Institute for Dementia-Related Diseases (Wroclaw-Poland) of the Medical University of Wroclaw. MCI participants were enrolled in this study after detailed clinical and neuropsychological examination, according to the NIA-AA criteria [21]. The exclusion criteria consisted of (1) the presence of visual and/or communication problems that could interfere with the study procedures; (2) illiterate participants, as it would hinder the administration of neuropsychological tests; (3) comorbidities such as cancer, severe renal or hepatic insufficiency, and severe cardiac or respiratory failure; (4) excessive alcohol intake (>280 g/week); (5) Computerized Tomography or Magnetic Resonance Imaging (MRI) evidence of hydrocephalus, stroke, a space-occupying lesion, or any clinically relevant central nervous system disease apart from AD; (6) the presence of mental disorders according to DSM-5-TR criteria; (7) the presence of untreated (or treated for less than 3 months before the screening visit) vitamin B12 or folate deficiency; and (8) the presence of untreated thyroid disease [25].

The patient, the responsible caregiver, and the legal representative (when different from the responsible caregiver) all provided written informed consent to participate in the study. Additionally, the participants underwent a neuropsychological evaluation and a CSF lumbar puncture. The ability to generate memory strategies was assessed using the TMS.

Finally, from the original cohort, only 47 participants (24 females and 23 males) had available and valid data regarding our main variables of interest (CSF markers and neuropsychological assessment), making up our final sample (25 Polish/22 Spanish). The sample was categorized into two distinct groups based on CSF values (see a detailed description in Section 2.2.2): normal CSF biomarkers, named CSF − (67.38 ± 10.81 years old and 11.00 ± 3.41 years of schooling), and possible AD, named CSF + (69.13 ± 9.73 years old and 10.97 ± 4.56 years of schooling). 

### 2.2. Cerebrospinal Fluid (CSF) Acquisition and Analysis

#### 2.2.1. CSF Determinations

The CSF biomarker variables include Aβ42, t-tau, and p-tau levels measured in ng/L. CSF samples were collected between 8:00 and 10:00 a.m. to avoid variations related to the circadian rhythm [26]. The samples were collected in polypropylene tubes, centrifuged at 2000× *g* for 10 min at 4 °C, immediately frozen, and stored within 4 h in a −80 °C freezer. Later, they were used for biomarker analysis. The biomarker variable levels were determined by the enzyme immunoassay method (ELISA) according to the manufacturer’s instructions. In this sense, the concentrations of Aβ-42, t-tau, and p-tau were measured using the following ELISA commercial kits (in Spain: Innotest β-Amyloid 1-42 for Aβ-42; Innotest hTAU Ag for t-tau; and Innotest Phospho-TAU181P for p-tau, Fujirebio-Europe, Gent, Belgium); (in Poland: ELISA kits for Aβ-42, IBL International, Hamburg, Germany; Innotest hTAU Ag for t-tau; and Innotest Phospho-TAU181P for p-tau, Fujirebio-Europe, Gent, Belgium). All the samples were measured in duplicate, and the values were expressed in ng/L. Finally, samples were obtained with support from IRBLleida Biobank (B.0000682) and PLATAFORMA BIOBANCOS PT17/0015/0027 [27].

#### 2.2.2. Classification Based on CSF Cut-Off Levels

Each CSF measure was dichotomously classified (i.e., positive or negative for AD) according to the following cut-off values: for t-tau and p-tau, the cut-off scores used were the ones proposed by Sjögren et al. [28] for Innotest ELISA assays (t-tau ≥ 450 ng/L, and p-tau ≥ 61 ng/L), which were the same to those reported in other previous and recent multicenter studies [29]; the cut-off value for Aβ42 was ≤500 ng/L according to Innotest [28] and IBL International scores. It is necessary to clarify that the measurements of CSF biomarkers in both, the Spanish and Polish participants, closely align with the aforementioned cut-off points. Subsequently, the sample was divided into five categories based on the Erlangen Score Diagnostic Algorithm (ESA) classification, which reflects the continuum between the entirely normal and the entirely pathological CSF measures: 0 points if all the CSF biomarkers are normal; 1 point reflects a pattern with marginal alterations in only one of the biomarkers (Aβ or tau, but not both); and 2 points establish a clear pathological CSF alteration in either Aβ metabolism (decreased Aβ42 concentrations and/or decreased Aβ42/40 ratio) or tau metabolism (increased concentrations of t-tau and/or p-tau) but not both. On the other hand, a clear alteration in one biomarker group (either Aβ or tau) with marginal alterations in the other group is scored with 3 points, and up to 4 points are considered when clear alterations were detected in both Aβ and tau/p-tau results [30]. Lastly, and considering the characteristics of our sample, we combined the obtained ESA categories as follows: those participants who had 0 or 1 points were classified as CSF − (“neurochemically improbable AD”), while those who had 2 or 3 points were categorized as CSF + (“neurochemically possible AD”).

### 2.3. Test of Memory Strategies (TMS)

TMS is an immediate verbal memory test in which, through five consecutive conditions, the necessity of using executive functions is progressively reduced. Each list includes 10 words that have been randomly selected based on their linguistic frequency. The TMS-1 is an incidental learning task with neither a semantic nor a phonetic relationship between the words, in which the participants are not aware if they are in the context of a memory task. TMS-2 to TMS-5 are explicit learning tasks, with a progressive organization of material into semantic categories. Every list/condition reduces the necessity for memory strategies and, therefore, the recruitment of executive functions.

In the cases of TMS-2 and TMS-3, the participants exhibit a greater need to implement internal recall strategies. Specifically, in TMS-3, the words should be recalled in groups based on two different semantic categories (with a low organization of the material), resulting in a greater requirement for activation of the working memory. Conversely, in TMS-4 and TMS-5 conditions, the lists of words are grouped and presented in two differentiated semantic categories (i.e., sports and vegetables), reducing the need for internal cognitive strategies due to the external organization of the material.

The words on every list are read at a rate of one word per second. Each correct answer receives one point, and the score varies from 0 to 10 for each list independently, or from 0 to 50 for the total scale, which refers to the sum of the five tasks/lists of words. 

### 2.4. Statistical Analyses

The descriptive statistical examination was performed using central tendency and dispersion analyses. The association between demographic, clinical, and neuropsychological data was evaluated by using Spearman’s correlation. On the other hand, and as already mentioned, the participants were divided according to the reference values in healthy individuals suggested by Sjögren et al., [28] and the ESA, validated by Lewczuk et al., [30], which describe the pattern of the Neurochemical Dementia Diagnostics (NDD) biomarkers and distinguish between CSF − (no evidence for organic Central Nervous System disease) and CSF + or possible AD (clearly pathological results of either Aβ or tau/p-tau, or both). Since the data were non-normal and heteroscedastic according to the Kolmogorov–Smirnoff and Levene tests, we used non-parametric contrast tests (Mann–Whitney U) to compare the groups. Finally, an analysis of covariance (ANCOVA) was performed to determine the effect on ESA classification in the sample, using the country of origin of the participants or, alternatively, their spoken language (i.e., Spanish or Polish) as a covariate. All the procedures were executed utilizing the IBM SPSS statistical package 22.0.

## 3. Results

### 3.1. Correlation between CSF Markers and TMS

When analyzing the correlation among demographic, clinical, and neuropsychological variables, it was observed the concentrations of CSF biomarkers did not correlate with age, years of education, or any of the four conditions of the TMS. Only significant associations between TMS-3 and Aβ42 (rho = 0.357; *p* < 0.05), t-tau (rho = −0.307; *p* < 0.05), and p-tau (rho = −0.337; *p* < 0.05) were observed for the whole sample (see Table 1).

Moreover, when dividing the sample into CSF − and CSF +, the difference between groups remained significant for the TMS-3 condition (*p* < 0.001) (see Table 2).

Table 3 presents the ANCOVA results based on the two classification criteria mentioned above, namely CSF − and CSF+, with the country of evaluation as a covariate. We observed statistically significant differences between groups in TMS-3 (F (1, 44) = 17.51, *p* < 0.001, partial η^2^ = 0.285). According to the ESA categorization, the participants with normal values for CSF biomarkers had a higher cognitive performance in TMS-3 compared to those in the possible AD group. These outcomes persisted if the groups were divided according to Aβ42 scores (*p* < 0.05), but not concerning t-tau (*p* = 0.50) and p-tau values (*p* = 0.46).

We further examined the effect of the interaction between CSF biomarkers and the country of origin on cognitive performance. The results were only significant in the case of fluency tests in tau (*p* < 0.05) and p-tau (*p* < 0.05). Lastly, the *p*-values were corrected by FDR (See Supplementary Materials).

### 3.2. Other Significant Correlations of Interest

Besides TMS and CSF correlations, we found additional interesting associations among age, years of education, TMS, and verbal fluency. We found a negative correlation between age and years of education (rho = −0.626; *p* < 0.01) and between age and TMS-1 (−0.367; *p* < 0.05). Furthermore, TMS-1 was positively associated with years of education (rho = 0.459; *p* < 0.01). TMS-2 presented a positive correlation with verbal fluency (rho = 0.356; *p* < 0.05). For additional details, please refer to Table 1.

## 4. Discussion

The present study aims to establish significant relationships between CSF values (i.e., Aβ-42 concentrations, t-tau, and p-tau) and TMS performance in a sample of MCI patients. TMS uses the episodic memory–executive functions axis to discriminate the origin of cognitive dysfunction in the early stages of AD. 

Regarding the TMS and its neuropsychological implications, the participants were enrolled in an immediate memory test, which included a total of five lists of words. In the first condition, the words lacked any semantic or phonological association, whereas in the last condition, the words were organized into two distinct semantic categories, such as sports or vegetables. Thus, the process begins with a higher reliance on memory strategies for encoding and retrieval, heavily dependent on executive functions, to an external organization of the material that requires fewer complex cognitive strategies. That is, the last conditions were focused on assessing the primary capacity for episodic memory. As a result of this type of test, the possibility of overlapping between memory impairment and executive dysfunction, which might hinder an accurate differential diagnosis from normal to pathological aging, could be solved. Additionally, its application has demonstrated adequate discriminant power to distinguish among various diagnostic groups [13,14].

For the whole MCI sample, positive (Aβ-42) and negative (t-tau and p-tau) correlations with the TMS-3 condition were observed. Specifically, the lower the concentration of Aβ-42 and the higher the tau levels, the lower the score in the TMS-3-word list. Moreover, upon stratifying participants according to the ESA classification, the outcomes revealed worse performance in the TMS-3 condition for the CSF + group, largely correlated with lower Aβ-42 CSF values. As described in the introduction, it is well known that there is interest in combining CSF values and neuropsychological scores as a useful approach for early AD detection, discrimination, and the monitorization of its progression [18,19,24]. 

**Figure 1:**
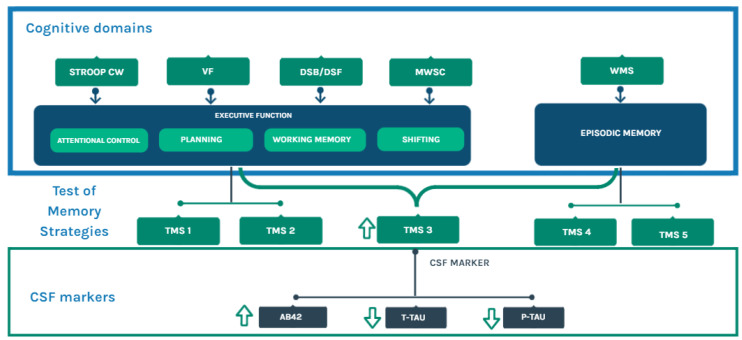
A review of cognitive functions that are associated with the Test of Memory Strategies (TMS) and their link to CSF biomarkers. TMS is an immediate verbal memory test with 5 consecutive conditions. In each condition, the use of the executive function is progressively reduced; TMS-3 is the word list condition where executive functions and episodic memory could be mainly combined. Interestingly, better performance in TMS-3 is associated with higher levels of Aβ42 and lower levels of t-tau and p-tau—classical biomarkers of Alzheimer’s Disease (AD). As described in the text, this scenario could lead us to hypothesize that our CSF + participants might be at a very prodromal stage of dementia. The neuropsychological components (specifically those pertaining to executive functioning and episodic memory) depicted in the figure, and their relationship with the TMS, derive from previous studies conducted by Fernandes et al. [14], Vaccaro et al. [15], and Abellán-Martínez et al. [31]. Stroop CW: Stroop Color and Word Test; VF: verbal fluency; DSB: digit span backward; DSF: digit span forward; MWSC: Modified Wisconsin Card Sorting Test; WMS: Weschler memory scale.

In the ESA-based group comparison, both MCI groups seem to perform cognitively similarly across TMS conditions, except for TMS-3. That could mean that executive and memory functions are impaired similarly in the possible AD (CSF +) and the other MCI patients (CSF −). Therefore, it would be challenging to distinguish them using conventional neuropsychological scores, despite the distinct pathophysiological profiles that have been determined through CSF biomarkers. The key point here is that TMS-3 appears to be sensible and sensitive enough to discriminate between patients with cognitive similarities and different pathophysiological profiles. For a clearer explanation of the TMS results and their relationship to CSF measures and other executive function/episodic memory tests, please see Figure 1. 

In TMS-3, the two semantic categories included are disorganized and require the subject’s intrinsic organization to successfully execute this condition, indicating the implementation of a cognitive strategy. Subjects with executive dysfunction, such as vascular patients, perform worse on this task [13]. The group differences disappear when an external organization is involved, as reflected in TMS-4 and 5 conditions. Additionally, it has been observed that in the case of patients with a primary memory deficit such as those with amnestic MCI, the increase in the external organization of the material did not improve their performance. However, patients with multidomain MCI obtained improved scores as the material was progressively organized [13]. This could indicate that executive function deficits are playing a much greater role in the performance of episodic memory tasks than previously thought. As stated by Abellán Martínez et al. [31], the typical reduction of cognitive performance in memory tasks throughout the aging process, particularly in those participants with possible AD, may stem from executive function impairment rather than primary memory deterioration.

Similar to our study, van der Vlies et al. [24] established clusters based on CSF biomarkers and their association with diverse cognitive profiles in a cohort of patients with AD. They reported that, depending on the CSF concentrations, patients presented a severe or minor cognitive impairment which could not be explained by disease severity. For instance, the patients with low levels of Aβ-42 and extremely high CSF levels of tau and p-tau (cluster 3) exhibited worse memory performance, mental speed, and executive functioning. However, the participants belonging to cluster 1, with lower abnormal biomarker values, showed less impairment of naming and memory. Thus, cluster 1 may be more comparable to our sample based on the CSF marker values and the findings of the correlation with cognitive performance. Specially, the outcomes regarding the Aβ-42 measures hold significant importance in our research, as the disparity observed between groups in TMS-3 is essentially correlated with lower Aβ-42 CSF values. 

Furthermore, amyloid plaques are commonly located in the prefrontal and parietal regions, causing disruption to the fronto-parietal networks traditionally associated with executive functioning [32,33]. Hence, it is logical to speculate that lower values of Aβ-42 in CSF may indicate a greater accumulation of amyloid in these cortical fronto-parietal regions, thereby causing difficulties in the performance of a demanding verbal learning task such as TMS-3. Indeed, the results of factor analyses have consistently revealed a strong association between the TMS-3 condition and executive functioning [14,31]. Furthermore, it should be noted that the TMS-3 condition requires active mobilization of the patient’s own cognitive strategies to achieve successful performance, underscoring the pivotal role of executive dysfunction in the execution of episodic memory tests among MCI patients. Moreover, it could also indicate that although the prefrontal cortex and, consequently, executive functioning is progressively affected by age [34,35], its early impairment in the dementia process has not been adequately addressed. 

On the other hand, TMS-3 correlates as well with tau and p-tau values. While tau has been primarily associated with the medial temporal lobe, amyloid is known to be spread throughout the entire cortex including the anterior cingulate and parietal regions. It has been proposed that tau proteins are prone to brain regions where amyloid deposits have already been established, linking the network of the proteins [36]. This could potentially account for the correlation between the TMS-3 condition and both proteins at the MCI stage. It would be pertinent to investigate whether a comparable correlation exists in the earlier stages of the disease. 

These findings suggest that the TMS could serve as a non-invasive and appropriate neuropsychological tool for identifying the primary source of cognitive dysfunction in MCI patients. It is still necessary to determine whether these cognitive changes are simply a product of a particular stage of the disease or if they are already present in preclinical stages, as well. Additionally, the fact that TMS correlates with current biomarkers makes it more suitable for clinical use, especially in patients in the process of AD. Episodic memory and executive functions are the two cognitive domains that are mainly affected in AD [37] and are also highly effective cognitive predictors for the onset of dementia in MCI patients [38,39]. Therefore, the ability of TMS to discriminate between them in a single test could reinforce the notion of considering this test a valuable and reliable instrument that might be applied in the very early stages of AD diagnosis.

Finally, it is noteworthy to mention the other significant correlations between specific word lists in the TMS and the verbal fluency task or demographic data relative to our sample. Of special interest are the positive correlations identified between TMS-2 and verbal fluency, as well as between TMS-1 and years of education. Furthermore, we observed a negative correlation between TMS-1 and age. It is generally accepted that verbal fluency is closely related to executive functioning [40]. Like the TMS-3, the TMS-2 is another condition of this test that is strongly related to the recruitment of executive functioning. Hence, the positive association between verbal fluency and TMS-2 supports and reinforces this relationship. In the same vein, the negative correlation between TMS-1 and age and the positive one between TMS-1 and years of education, together with the lack of significant differences between groups in TMS-1 performance, could emphasize the role of cognitive reserve and its importance against brain damage [41,42,43,44], specifically, to cognitive processes (e.g., episodic memory and executive functions) that, as has been exposed, are more vulnerable to the aging process [31,45,46,47]. 

### Limitations and Future Directions

One of the main weaknesses in the present investigation may be related to the ongoing validation process of the Polish version of the TMS. Despite this, the preliminary findings are quite robust. The length of words, phonology, pronunciation, semantic category, or frequency of use (especially in the word-list tests) could be critical in the way the words are organized for encoding and retrieval [48,49]. Nevertheless, in the ANCOVA analyses, to contrast the performance in the TMS between the CSF + and CSF − participants, we introduced the country of origin/spoken language as a covariate with the intention of minimizing the impact of this confounding factor. Likewise, besides language, the effects of age and education on test performance have been widely acknowledged for several decades [50,51,52]. Bearing this in mind, these two variables were controlled.

Another important issue could be the scarcity of shared neuropsychological tests between the Polish and Spanish participants (i.e., we only had CSF biomarker measures, the TMS, and verbal fluency scores). Despite this, and in light of the results shown by Schindler and co-workers [20], this might not be a disadvantage given the characteristics of our sample. Furthermore, besides the Spanish version of the TMS [13,31], correlations with classic neuropsychological tests in other recently validated versions, such as the Portuguese [14] or Italian [15], support good convergent and discriminant validity, as well as adequate internal consistency and reliability for the three languages. In any case, future studies should try to incorporate a similar multicenter neuropsychological battery to test other potential influences of languages and cultures.

Finally, in line with the CSF cut-off levels used to distinguish AD patients from healthy controls [28], and considering that the different diagnostic categories established by Engelborghs and co-workers [19] are supported by the reported CSF biomarker profiles, it is important to contemplate that although our MCI participants might be in a very prodromal disease stage (i.e., with higher Aβ-42 and lower t-tau and p-tau levels), their CSF biomarker profile may not match the conventional pattern seen in AD (i.e., lower Aβ-42 values and higher t-tau and p-tau levels). In other words, it is possible that their progression may not ultimately culminate in a “pure” or “typical” manifestation of AD.

On the contrary, despite the existence of numerous studies demonstrating the correlation between Aβ levels in CSF and PET amyloid, a PET amyloid study has not been performed on this occasion. This means that we cannot draw conclusions about the location of the Aβ plaques and their correlation with the neuropsychological deficits of the patients. In the same way, for a more comprehensive assessment of amyloid pathology, it would have been more appropriate to analyze the ratio of Aβ-42/Aβ-40 rather than solely focusing on the Aβ-42 levels. However, due to a technical resource constraint, we were unable to execute this more precise analysis.

Additionally, our CSF + group consisted of a relatively small sample size of 31 participants with MCI. Despite the high clinical value of the CSF data included in the study, our results should be considered preliminary. Therefore, forthcoming research studies would necessitate larger sample sizes focusing on the identification of meaningful subtypes of the disease (i.e., multidomain MCI or vascular cognitive impairment). To accomplish this objective, it is imperative to conduct thorough follow-ups to better characterize the recruited participants.

## 5. Conclusions

Our study reveals the correlation between the TMS-3 condition of the TMS and typical CSF biomarkers of AD. Considering that this condition is associated with the requirement of executive functioning, this fact could indicate an early executive functioning impairment in MCI patients, which could ultimately affect their performance on episodic memory tests (verbal learning lists). This could have important implications not only at the neurochemical level, given the possibility of replacing the use of an invasive technique such as the lumbar puncture, but also at the neuropsychological level when distinguishing different subtypes of MCI. Therefore, this could lead to more precise/personalized rehabilitation programs. It is generally recognized that the primary needs of patients with MCI are episodic memory training interventions. Nonetheless, our findings exhibit the necessity of supplementary and prompt intervention in the executive functioning process, as it may have an impact on the participants’ capacity to encode and organize the information in their memory. Future studies should address how executive training in MCI patients improves the scores on TMS-3 as well as in daily living activities.

## Figures and Tables

**Table 1:** Association between demographic, clinical, and neuropsychological data.

	Age	Years of Education	AB 42	Tau	p tau	TMS 1	TMS 2	TMS 3	TMS 4	TMS 5	Fluency Test
Age	1	−0.626 **	0.050	0.014	0.192	−0.367 *	0.140	0.141	0.163	0.145	0.276
Years of education		1	−0.005	0.102	−0.027	0.459 **	−0.160	−0.210	−0.085	−0.158	−0.091
AB 42			1	−0.228	−0.325 *	0.257	0.234	0.357 *	0.258	0.101	0.189
T-Tau				1	0.869 *	0.173	−0.205	−0.307 *	−0.151	−0.066	−0.198
P- tau					1	0.000	−0.125	−0.337 *	−0.090	−0.040	0.010
TMS 1						1	0.322 *	0.113	0.077	0.137	−0.069
TMS 2							1	0.539 **	0.490 **	0.583 **	0.356 *
TMS 3								1	0.499 **	0.648 **	0.154
TMS 4									1	0.397 **	0.244
TMS 5										1	0.284
Fluency Test											1

** The correlation is significant at the 0.01 level (bilateral). * The correlation is significant at the 0.05 level (bilateral).

**Table 2:** Socio-demographic and neuropsychological measures divided by ESA classification.

Variable	Mean ± SD	CSF – (*n* = 16)	CSF + (*n* = 31)	*p*-Value *
Age	68.53 ± 10.03	67.38 ± 10.81	69.13 ± 9.73	0.701
Years of education	10.98 ± 4.17	11.00 ± 3.41	10.97 ± 4.56	0.701
TMS 1	4.30 ± 3.17	5.06 ± 3.28	3.90 ± 3.09	0.308
TMS 2	2.47 ± 1.36	3.00 ± 1.59	2.19 ± 1.17	0.166
TMS 3	3.15 ± 1.50	4.25 ± 1.18	2.58 ± 1.34	**<0.001**
TMS 4	3.72 ± 1.77	4.25 ± 1.24	3.45 ± 1.95	0.166
TMS 5	4.53 ± 1.82	5.25 ± 1.77	4.16 ± 1.75	0.166
Fluency test	8.15 ± 3.63	9.00 ± 4.08	7.71 ± 3.37	0.591

Note. We present values as mean ± standard deviation (SD) for the characteristics of the participants as well as the variables used for correlation analyses. These include age (in years), years of education, and Test of Memory Strategy (TMS). The Mann–Whitney U test was used to compare within-group differences. * *p*-value corrected by FDR at 5%.

**Table 3:** ESA classification and performance in TMS.

Factor	F-Statistic	*p*-Value *	Effect Size
TMS 1	3.9	0.087	0.081
TMS 2	3.78	0.087	0.079
TMS 3	17.51	<**0.001**	0.285
TMS 4	2.11	0.184	0.046
TMS 5	3.87	0.087	0.081
Fluency Test	1.23	0.274	0.027

Note. Statistical results of the ANCOVA used the country of origin as a covariate. Effect size is reported as partial η^2^. F-statistic, F (1, 44). * *p*-value corrected by FDR at 5%.

## Data Availability

The data presented in this study are available on request from the corresponding author.

## Supplementary Materials

The following supporting information can be downloaded at: https://www.mdpi.com/article/10.3390/biomedicines11123147/s1, Table S1: Interaction between CSF biomarkers classification and country of origin and the effect in TMS scores.
