# Supplementary material for: Understanding the Episodic Memory and Executive Functioning Axis Impairment in MCI Patients: A Multicenter Study in Comparison with CSF Biomarkers
Source: Biomedicines. 2023 Nov 26;11(12):3147. doi: 10.3390/biomedicines11123147 (PMC10741228; doi:10.3390/biomedicines11123147)
Supplement: Supplementary file 1 [file biomedicines-11-03147-s001.zip › biomedicines-2665882-supplementary.pdf]

Supplementary Material

S1. Interaction between CSF biomarkers classification, country of origin and the effect in TMS scores

Aβ42*country of origin

**Factor**	**F-statistic**	***p-value****	**Effect size**
TMS 1	1.138	0.650	0.026
TMS 2	.378	0.650	0.009
TMS 3	.004	0.953	0.000
TMS 4	.385	0.650	0.009
TMS 5	.483	0.650	0.011
Fluency Test	5.398	0.150	0.112

t-tau*country of origin

**Factor**	**F-statistic**	***p-value****	**Effect size**
TMS 1	1.048	0.818	0.024
TMS 2	0.054	0.818	0.001
TMS 3	0.071	0.818	0.002
TMS 4	0.056	0.818	0.001
TMS 5	0.060	0.818	0.001
Fluency Test	10.848	0.012	0.201

p-tau*country of origin

**Factor**	**F-statistic**	***p-value****	**Effect size**
TMS 1	.004	0.950	.000
TMS 2	.281	0.950	.006
TMS 3	.076	0.950	.002
TMS 4	.306	0.950	.007
TMS 5	.011	0.950	.000
Fluency Test	12.438	0.006	.224

Note. Statistical results of the ANCOVA used the country of origin as a covariate. Effect size is reported as partial η^2. F-statistic, $F(1, 44)$. **p-value* corrected by FDR at 5%.
